# Health professionals’ knowledge and attitudes to healthcare-seeking practices and complementary alternative medicine usage in Ugandans with diabetes: a cross-sectional survey

**DOI:** 10.11604/pamj.2017.28.256.11615

**Published:** 2017-11-22

**Authors:** Fortunate Atwine, Katarina Hjelm

**Affiliations:** 1 School of Health and Caring Science, Linnaeus University, Vaxjo, Sweden; 2 Department of Nursing, Mbarara University of Science and Technology (MUST), Mbarara, Uganda; 3 Department of Social and Welfare Studies, University of Linkoping, Campus Norrkoping, Sweden

**Keywords:** Attitudes, complementary and alternative medicine, Diabetes, healthcare providers, healthcare-seeking behaviour, knowledge

## Abstract

**Introduction:**

Healthcare-seeking behaviour among persons with diabetes has been investigated to a limited extent, and not from professionals’ perspective. The aim of the study was to describe healthcare professionals’ knowledge, attitudes and practice concerning healthcare-seeking behaviour and the use of complementary and alternative medicine among persons with diabetes.

**Methods:**

A cross-sectional, self-administered questionnaire was conducted in western Uganda. Nurses, midwives or nurse assistants 72.2%, physicians 12% and clinical officers 10% volunteered to participate in the study with a total 108 (93% response rate) response rate. Descriptive statistics were used to analyse data with frequencies, percentages and summarized in tables.

**Results:**

Most of the healthcare providers perceived more uneducated people to be at risk of developing complications related to diabetes (66.7%) and that most of the patients with diabetes were not knowledgeable about signs and symptoms of diabetes before being diagnosed (75.9%). The main reasons inducing persons with diabetes to seek care outside the health care sector were reported to be seeking a cure for the condition, influence from the popular sector, the accessibility of the place and signs of complications of diabetes related to poor glycaemic control. Healthcare providers had relatively positive attitudes towards using complementary and alternative medicine.

**Conclusion:**

Insufficient knowledge about diabetes, compromised healthcare-seeking practices including drug procurement for diabetes seem to be barriers to diabetes management. Patients were thus reported to be burdened with co-morbidities of complications of diabetes related to poor glycaemic control.

## Introduction

Healthcare-seeking behaviour among persons with diabetes mellitus has been investigated to a limited extent in developing countries, with a few exceptions [1-3]. A switch between different health care providers may negatively affect diabetes management and health [4]. While medical management is known for diagnosis and glycaemic control in diabetes care [5], health professionals’ knowledge is lacking about healthcare-seeking practices and attitudes to the use of complementary alternative medicine to manage diabetes. Examining consumers’ healthcare practices from the health professionals’ perspective can help in developing strategies to ensure better access to care for effective health management. Healthcare can be sought from different sectors in society: the popular sector among family, friends and relatives; or the professional sector among health professionals; or traditional healers in the folk sector [6]. Traditional medicine (TM) is either the mainstay of healthcare delivery or serves as an adjunct; in some countries it is termed complementary and alternative medicine (CAM) [7]. Healthcare-seeking behaviour with a switch between different healthcare providers under the influence of the popular and folk sector has been reported from the perspectives of the persons with diabetes [1, 2] and traditional healers [3]. Perceived failure of the effect of treatment of diabetes from the professional sector and self-care deficit with persisting symptoms related to diabetes were the main reasons [1-3].

Nurses play a key role by acting as coordinators in organizing holistic care to meet patients’ needs based on individual beliefs about health and illness [1]. Patients’ expectations, health beliefs and values should be integrated into the patients’ care process [8]. Perceiving some relief of disease symptoms by using CAM along with diet control and exercises indicates a maximum degree of diabetes management satisfaction [9]. Health professionals need to note that people with diabetes mellitus are likely to continue switching between different healthcare providers to manage diabetes because of community influence and perceived beliefs that CAM cures, is affordable and easily accessible [3].

The lifelong process of treatment for diabetes mellitus can be burdensome and expensive for the patients, consequently leading the national healthcare system to be overwhelmed by extra costs [10]. The socio-economic burden of diabetes treatment has a substantial impact, forcing many persons with diabetes to switch between different healthcare providers and use CAM as a less expensive alternative measure to manage diabetes and its complications [2, 3]. Diabetes is a common global metabolic disorder and the prevalence of diabetes in adults has been increasing in recent decades [11]. Since diabetes mellitus is growing into a pandemic, particularly increasing in Sub-Saharan Africa, the prevalence of diabetes-related complications is likely to increase [12]. Uganda, by virtue of the large existing population, appears among the first 10 African countries in the region with the largest number of people living with type 2 diabetes (i.e. exceeding 1.5 million individuals) [13].

Diabetes care in Uganda is run in the general healthcare system of public and private facilities [14]. Some of the hospitals in Uganda have established outpatient diabetes clinics. These operate once weekly across the country in public hospitals. There is no national health insurance system. The government is the main provider of health services free for the users, but health services are underfunded and frequently drugs are not available. Besides formal organization in the professional health sector, there are also traditional and complementary medicine practitioners [15]. Perceived failure in healthcare to manage diabetes or related complications leads many people in Uganda, particularly women, to seek CAM in the folk sector [1]. One of the challenges of CAM therapies in developing countries is that most of the medicines bear labels without known chemical names [3] and this makes it difficult to share the information with the healthcare professionals for effective case management. CAM usage is acknowledged and growing widely in both low and high income countries and the demand for its services is increasing [7].

Despite the use of CAM in many conditions, little is known about the proficiency of nurses [16] or other healthcare professionals regarding how it is used to manage diabetes mellitus in combination with western medicine. Previously some studies on knowledge and attitudes to CAM investigated CAM usage among healthcare providers without disease specificity and healthcare seeking practices of patients [17-19] though other studies have included the ability of healthcare staff to communicate the risks and benefits of CAM to patients [20, 21]. Before considering the integration of CAM into the professional healthcare system there must be sufficient knowledge, willingness, and receptivity among professionals to deliver or promote the administration of certain CAM therapies [17]. This includes a medical follow-up package of a comprehensive health assessment that proceeds to individualized psychological educational and clinical support of the patient to sustain long-term adjusted behaviour in order to achieve self-care management [22] of diabetes. If health professionals are to achieve the above objective, they need first to understand that healthcare-seeking practices include switching between different healthcare providers [1,2]. Living conditions of the people in the study area plus treatment cost, healthcare organization, individuals’ beliefs about health and illness and general condition seem to influence healthcare-seeking behaviour [3]. Therefore, as advocates working for the well-being of patients, health professionals need to be aware of the dynamic activities of healthcare-seeking practices of patients in relation to CAM usage. The aim of the study was to describe health professionals’ knowledge, attitudes and practice concerning healthcare-seeking behaviour and the use of CAM among persons with diabetes.

## Methods

### Design

A cross-sectional descriptive study was conducted using a self-administered questionnaire to investigate knowledge, attitudes and practice of healthcare providers concerning healthcare-seeking behaviour and the use of CAM therapies in patients with diabetes. The design enabled the investigators to gather information on the variables of interest to describe diabetes management using CAM therapies from healthcare providers’ perspectives [23].

### Participants

All known healthcare providers working with management of diabetes in a certain region of south-western Uganda, including rural and urban areas, were invited to participate in the study. Respondents were representatives of a public regional referral hospital, two mission (not-for-profit) hospitals, a private hospital and five health centre IVs. Health Centre IV is a mini hospital serving 70,000-100,000 people for general services at a county level [15]. The strategy was to generate as complete information as possible about diabetes care at all levels of the health care system. All respondents were approached while on duty in their respective healthcare facilities, in outpatient, medical, surgical and emergency departments. The inclusion criteria were: all healthcare providers aged >18 years that freely consented, employees of that particular healthcare facility and literate in english. Exclusion criteria were intended to eliminate all healthcare providers working below health centre IVs, because these healthcare facilities do not provide diabetes care. The response rate was 93% (n=108).

### Data collection

Data collection ran from April to September 2015. A self-administered questionnaire was used to collect data of the respondents’ knowledge, attitudes, practice on healthcare-seeking behaviour of patients with diabetes and the use of CAM to manage diabetes. A registered nurse (first author) distributed the questionnaires to all health facilities and collected them when they were completed. If needed, clarification was made because she kept around to finish the procedure before moving to another health facility.

### Data collection instrument

**Figure 1:**
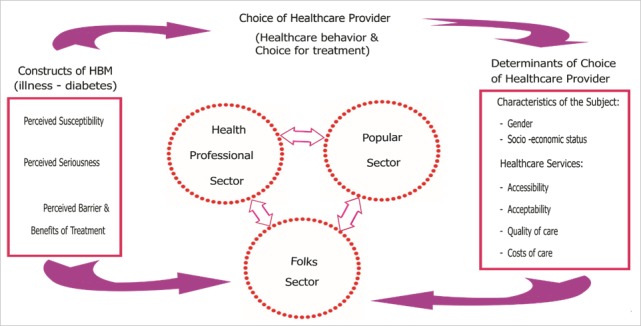
Pathways and determinants of choice of healthcare providers

A structured questionnaire with closed-ended questions was developed based on literature and previous studies [1-3, 8, 24], the health belief model [25] and the healthcare-seeking explanatory models [6, 26]. The theoretical framework with pathways and determinants of choice of healthcare providers is shown in Figure 1. The questionnaire covered background data, health professionals’ knowledge related to diabetes care, healthcare-seeking practices of patients with diabetes, health problems reported for those already on treatment and attitudes to CAM use. The responses to questions on diabetes care were ranked on a five-point Likert scale from “strongly agree” to “strongly disagree” and the mid-point was undecided. The section on healthcare seeking had multiple choices and responses were given by ticking only one option to show what they knew about the subject. Attitudes to CAM use were assessed by a previously validated 10-item CAM Health Belief Questionnaire (CHBQ) because it was a practical and reliable instrument with a Cronbach’s coefficient alpha of 0.75 [8]. Items were framed in a seven-point Likert-type rating scale format (1 - “Absolutely disagree” to 7 - “Absolutely Agree”). Directions to the CHBQ were “Please read and respond to each of the 10 statements below by circling the number that most agrees with your belief. “Yes or No” questions were asked to determine whether health professionals recommended CAM therapies. If the answer was yes, each respondent was asked to mark therapies that she/he had recommended to patients during previous experience. The whole questionnaire was peer-reviewed by nurses and general practitioners working in diabetes care. Face and content validity was tested by a pilot test [23] on four nurses and two doctors (not included in the study) from one of the hospitals before data collection, and minor corrections were made.

### Ethical considerations

Ethical approval was obtained from the Faculty Research Ethics Committee (FREC) and Institution Research Board (IRB) of the university in the region, the District Health Officer, and permission to collect data from the healthcare facility managers. Healthcare providers were assured verbally and via information forms of the anonymity and confidentiality of the information provided. All respondents gave written informed consent and research procedures were conducted in accordance with the principles in the Declaration of Helsinki [27].

### Data analysis

Descriptive statistics for variables were used and frequencies were shown in percentages, means and standard deviation and summarized in tables [28]. Analyses were performed using the Statistical Package for Social Science (SPSS) IBM SPSS software program (version 23.0).

## Results

### Characteristics of respondents and diabetes care

One hundred and eight healthcare providers were included in the study population, of whom 57 % were female (Table 1). The majority were nurses, midwives or nurse assistants but there were also physicians (12%), clinical officers (10%), and assistants in medical laboratories and pharmacies. The majority had a diploma or certificate while about one tenth had university education. The participants were rather young, with a mean age of 34 years, had limited experience of work at the present workplace (mean 5 years) and had worked about 9 years in the health sector. About two thirds stated that the time used for patient consultations for a physician or clinical officer was less than 30 minutes, and more than a half of the respondents did not recommend CAM therapies to the patients with diabetes.

**Table 1:** Characteristics of study population

Variables	Frequency n (%)
Participants’ age in years ^1^	34 (23-59)
Years at present workplace ^1^	5 (1–3)
Years of work in health sector^1^	9 (3–38)
**Gender (n)**	
Female	62 (57.4)
Male	46 (42.6)
**Current professional title (n)**	
Medical doctor	13 (12.0)
Clinical officer	11 (10.2)
Nurses (enrolled & registered)	63 (58.3)
Midwives (enrolled & registered)	11 (10.2)
Medical laboratory assistant	5 (4.6 )
Pharmacy assistant	1 ( 0.1)
Nursing assistant	4 (3.7)
**Level of education (n)**	
Secondary, vocational training	37 (34.3)
Post-secondary vocational training	57 (52.8)
University graduate	9 (8.3)
Masters in medicine/nursing	5 (4.6)
**Consultation time between the doctor/clinical officer of patients with diabetes**	
< 29 minutes	59 (60.6)
>30 minutes	39(38.5)
**Recommended CAM therapies to patients with diabetes (n)**	
Healthcare providers not willing to recommend CAM therapies	62 (57.4)
Healthcare providers willing to recommend CAM therapies	46 (42.6)

1 Mean (range)

### Knowledge of healthcare providers on diabetes care

Most of the healthcare providers perceived more uneducated people to be at risk of developing complications related to diabetes (66.7%, Table 2) and that most of the patients with diabetes were not knowledgeable about signs and symptoms of diabetes before being diagnosed (75.9 %). Based on their experience, they perceived healthcare workers to be familiar with signs and symptoms of diabetes (80.6%). They thought that patients would come for regular follow-up if told by the physician or clinical officer (85.2%) and that most patients who had sought care from them would be willing to disclose where else they had got treatment (66.7%).

**Table 2:** Knowledge of healthcare providers about diabetes care

Statements	Strongly agree n (%)	Moderately agree n (%)	Undecided n (%)	Moderately disagree n (%)	Strongly disagree n (%)
More uneducated people are at risk of developing diabetes related to complications than those who are educated	11(10.2)	23(21.3)	2 (1.9)	3(21.3)	49(45.4)
If doctor/clinical officer has told the patient with diabetes to come for regular follow-up the patient will come	27(25.0)	65(60.2)	5(4.6)	8(7.4)	3(2.8)
Based on your experience, you think most healthcare workers are familiar with signs and symptoms of diabetes	42(38.9)	45(41.7)	3(2.8)	16(14.8)	2(1.9)
Based on your experience, you would say that most of the patients with diabetes were knowledgeable about signs and symptoms of diabetes before they were diagnosed	6(5.6)	15(13.9)	5(4.6)	27(25.0)	55(50.9)
Most of the patients who seek treatment here are also willing to tell you where else they get care	6(24.1)	48(42.6)	8(7.4)	19(17.6)	9 (8.3)

### Knowledge of healthcare seeking patterns in diabetic persons from healthcare providers’ perspective

Healthcare providers mainly stated that persons with diabetes buy medicine from a drug shop or pharmacy and use home self-medication or search for help from private health facilities or from traditional healers, but to a lesser extent before they seek help from them (22.9 vs 12.4%, Table 3). In some cases persons with diabetes were also stated to seek help from general hospitals, health care centres on different levels and spiritual healers.

**Table 3:** Healthcare-seeking patterns as known to healthcare providers

Where people with diabetes seek care before they come to the health facility	n (%)	Where else patients go if the doctor/ clinical officer has told them to come and they fail to do so for regular follow-up	n (%)
Buy medicine from drug shop/pharmacy	27 (25.7)	Buy medicine from drug shop/pharmacy	36 (33.6)
Private health facility	24 (22.9)	Traditional healers	20 (18.7)
Home-self medication	23 (21.9)	Private health facility	18 (16.8)
Traditional healers	13 (12.4)	Home-self medication	18 (16.8)
General hospitals	8 (7.6)	Spiritual healers	10 ( 9.3)
Health centre IV	5 (4.8)	General hospitals	4 (3.7)
Health centre I	2 (1.9)	Health centre IV	1 (0.9)
Health centre III	1 (1.0)	Health centre III	0 (00)
Spiritual healers	1 (1.0)	Health centre I	0 (00)

If persons with diabetes perceive that the treatment they receive from health professionals has no effect, they will seek help, according to the healthcare providers’ perspective, also mainly by buying medicine from drug shops or pharmacies (33.6%), followed by consultation with traditional healers and to a lesser extent with private health facilities (18.7 vs 16.8%). However, to a higher extent than when the person had sought help before coming to them, they also believed they would consult spiritual healers (9.3 vs 1.0%).

The main reasons inducing persons with diabetes to seek care outside the health care sector, according to healthcare providers, were searching for a cure for the condition, influence from the popular sector in terms of friends, relatives or social media, perceived threat of the condition or accessibility to the place (Table 4). Signs and symptoms related to poor glycaemic control and complications related to diabetes (Table 5), such as feelings of pins and needles in the limbs due to diabetes neuropathy, general body weakness, excessive thirst, bodily pain in the abdomen, foot ulcers, high blood pressure, nephropathy and history of stroke were the conditions most commonly seen at the health facility among persons with diabetes as stated by healthcare providers.

**Table 4:** Reasons that induce patients to seek care elsewhere than from medical health facility

Statement	Frequency n (%)
Search for cure of the condition	94 (87.0)
Influence from popular sector: friends, relatives, social media	83(77.6)
Perceived threat of the condition	81 (75.7)
Place accessible	80 (74.8)
Perceived cost effectiveness	71 (67.6)
The health facility is very far from the patients	68 (63.6)
Persistent signs and symptoms related to poor glycaemic control	66 (61.7)
Pressure from co-morbidities, e.g. high blood pressure	62 (59.0)
Fear of unpleasant side effects of western medicine	61 (57.0)
Perceived failure of western medicine to improve general condition	58 (54.0)
Have a break from western medicine	58 (54.0)
Personally found it beneficial	56 (52.3)
Patients know therapist with good reputation	51 (48.1)

**Table 5:** Chronic conditions commonly seen at the health facility among patients with diabetes

Chronic conditions	Frequency n (%)
Diabetes neuropathy, pins and needle pain in the limbs	103 (95.4)
High blood pressure	100 (92.6)
General body weakness	100 (92.6))
Excessive thirst	97 (88.8)
Diabetes nephropathy	74 (68.7)
Foot ulcer	73 (67.7)
High cholesterol	65 (60.2)
Bodily pain e.g. abdomen	61 (56.5)
History of stroke	53 (49.1)

### Attitudes to use complementary and alternative medicine in healthcare providers

The healthcare providers had relatively positive attitudes to using CAM (Table 6). The maximum possible score of total number of responses on the questions was 70, with a hypothetical midpoint of 35 (denoting neutral attitude). The results in this study were 49.4 ±9.5 as a mean score.

**Table 6:** Beliefs about complementary and alternative medicine of healthcare providers

Statement	Mean	Standard deviation
Physical and mental health are maintained by an underlying energy or vital force	4.7	1.9
Health and diseases are a reflection of balance between positive life-enhancing forces and negative destructive forces	5.0	1.8
The body is essentially self-healing and the task of a healthcare provider is to assist in the healing process	5.3	1.7
A patient’s symptoms should be regarded as a manifestation of a general imbalance of dysfunction affecting the whole body	5.3	1.6
A patient’s expectations, health belief and values should be integrated into the patient’s care process	5.7	1.5
Complementary therapies are a threat to public health	4.1	1.9
Treatment not tested in a scientifically recognized manner should be discouraged	5.1	1.9
Effects of complementary therapies are usually the result of a placebo effect	4.5	1.6
Complementary therapies include ideas and methods from which conventional medicine could benefit	4.9	1.6
Most complementary therapies stimulate the body’s natural therapeutic powers	4.5	1.6
Total score	49.4	9.5

All items used a 7-point scale with 1=“Absolutely Disagree” and 7=“Absolutely Agree” Respondents to all CHBQ items were scored so that a higher response indicated greater endorsement and more positive attitude. CHBQ total scores were computed by summing across the 10 rating items. The maximum possible score of total number of responses to the 10 questions was 70 with a hypothetical midpoint of 35 (denoting neutral attitude). The computation result in this study was 49±9.5 as mean score.

In general less than half (42.6%) of the healthcare providers stated giving recommendations for using CAM therapies but to a limited extent (Table 1). The most frequently recommended types of CAM therapies were dietary supplements (27.8%, Table 7) followed by massage (18.5%), prayers (17.6%), dance and music (12.9 vs 11.1%). More traditional forms of CAM such as acupuncture, Chinese and herbal medicine were mentioned, but to a lesser extent (about 10 to 7%).

**Table 7:** Complementary and alternative therapies recommended by the healthcare providers

Type of complementary and alternative therapy	Frequency n (%)
Dietary supplements	30 (27.8)
Massage	20 (18.5)
Prayer	19 (17.6)
Dance	14 (12.9)
Music	12 (11.1)
Acupuncture	10 (10.2)
Chinese medicine	9 (8.3)
Herbal medicine	8 (7.4)
Osteopathic medicine	7 (6.5)
Visualization and guided imagery	4 (3.7)
Yoga	3 (2.8)
Kinesiology	3 (2.8)
Homeopathy	2 (1.8)
Qigong	2 (1.8)
Chiropractic therapy	2 (1.8)
Reiki	1 (0.9)
Ayurveda	1 (0.9)
Tai-chi	1 (0.9)

## Discussion

This study is unique as it focuses on the description of healthcare professionals’ knowledge, attitudes and practice of healthcare-seeking behaviour and the use of CAM among persons with diabetes mellitus in a limited resource setting. The main results showed that healthcare providers were knowledgeable about signs and symptoms of diabetes, unlike the patients who were reported to have insufficient knowledge on the subject. More than three quarters of healthcare providers stated that persons with diabetes reported back whenever they were given an appointment for a medical check up.

However, this gave contradictory information because when the person felt that expectations were not met or that treatment received from the professional health sector had no effect, there was a shift of healthcare-seeking practices to consulting traditional and spiritual healers. Self-medication and drug procurement were more prominent than before. Seeking a cure for the condition, being influenced by the popular sector, perceived threat of the condition or accessibility to the place were stated as reasons for seeking care elsewhere than from the professional health sector. Despite switching between different healthcare providers for patients with diabetes, several comorbidities still reported by more than three quarters of healthcare providers were signs or complications of diabetes related to poor glycaemic control. It was highlighted that healthcare providers were relatively positive towards CAM, however and just under half of them stated giving recommendation for their use, such as dietary supplements, massage, prayers, dance and music.

People with diabetes should receive medical care from a collaborative, integrated team with expertise in diabetes [5], because the role of the person with diabetes is crucial for diet, weight loss, physical activity, taking medication and because the involvement of multiple organ systems means that the care of patients often involves comprehensive services or specialties [29]. This strategy is far from the results of this study. There were discrepancies in the distribution of qualified personnel, especially at health centre IVs which had very few healthcare providers and also had the lowest level of education. Differences were found in distribution between nurses and midwives, 68.5% vs doctors and clinical officers 22%. A dissimilarity in this was that most nurses and midwives had a diploma or certificate nurses while doctors had university degrees and some had masters’ degrees in medicine. Clinical officers were also diploma holders who have tasks to perform in the general outpatient departments in the hospitals and are responsible for general medical services in health centre IVs. The lowest cadres were nursing and laboratory assistants who were found at health centre IVs. The primary health care system for non-communicable diseases is reported to remain weak in Sub-Saharan African [30] and most high-level health facilities in Uganda fail to meet minimal standards for interventions against non-communicable diseases [31].

A study in India reports that belonging to a relatively higher socio-economic status (SES) gives lower odds of having undiagnosed diabetes compared to poorer subjects because these subjects have the potential to access information and can afford better healthcare-seeking practices [32]. Some modifiable risk factors of diabetes were known to healthcare providers, such as low level of education of patients, insufficient knowledge of signs and symptoms of diabetes before diagnosis and compromised healthcare-seeking practices of people with diabetes. Underutilization of lower-level health facilities of non-communicable diseases (diabetes) is attributed to lack of guidelines, diagnostic equipment, drugs, lack of training and support supervision at these facilities, thus increasing the high burden of diabetes and hypertension cases to be managed at the hospitals in Uganda [33]

Patients who come to the health facilities would benefit from a diabetes self-management education package, i.e. follow-up care, tests, medication management, education, behavioural goal setting, psychosocial support [22]. However, < 30 minutes stated as consultation time between the patient and the doctor/clinical officer is very short to permit effective diabetes care as required. The above practice is contrary to the care offered by CAM therapists in the folk sector, who seem to be committed to their job, working through patient assessment, diagnosis, teaching, including counselling, providing medicine and monitoring patients even when they are at home [3].

The complexity of diabetes related to care seeking is a multifactorial interplay between genetic, e.g. individual characteristics, knowledge, attitudes, patients’ health belief and environmental influences such as costs, community setting plus healthcare organization, which together form summative pathways for healthcare-seeking practices [6, 25, 26]. Lack of a clear referral system in the health organization in Uganda leaves referral hospitals overcrowded, leading to poor case management [15]. Healthcare-seeking practices of these patients were described to involve back-and-forth movement including procurement of medication, and therefore the lines between the popular, folk and professional health sectors are porous; they thus act as points of entry and exit of patients at any time [6].

From a theoretical perspective in this study, reasons for care seeking give an impression that patients are ready to act because they are searching for a cure [25] but under the influence of the popular sector [6] since there is threat to their condition [25]. Accessibility and cost effectiveness are considered to some extent [26]. Fear of unpleasant side effects of western medicine, having a break from western medicine and perceived failure of western medicine to improve health are also noted by healthcare providers [6]. This provides a platform for many countries to recognize the need to develop a cohesive and integrative approach to healthcare that allows healthcare practitioners and, importantly, consumers of healthcare services to access CAM in a safe, respectful, cost-efficient and effective way [7].

Diabetes and hypertension make up 92% of the referral cases from health centre II (HCII) and health centre III (HCIII) in Uganda [33]. Those with more signs of diabetes match the co-morbidities of diabetes reported by the healthcare providers in this study, such as diabetic neuropathy, polydipsia, malaise, foot ulcer, which are true presentation of signs or complications of diabetes related to poor glycaemic control [2, 3].

Studies on knowledge, attitudes and practice regarding CAM show a low level, as healthcare providers had no previous education on the subject but express interest to learn about it [17, 18]. However, this objective is beyond the scope of this study. It was observed that healthcare providers were relatively positive towards CAM. The highest response scored means were 5.7 SD ± 1.5 on the attitudinal statement that patient expectations, health beliefs and values should be integrated into the patient’s care process. This indicated an endorsement of a positive attitude towards CAM [8]. While other healthcare providers recommended massage, dietary supplements and prayers, as the most common CAM therapies [20,21]; known and unknown local herbal extracts, nutritional products and counselling are the CAM therapies frequently offered to patients with diabetes from the folk sector [2, 3].

### Study limitations

All known healthcare providers, including urban and rural areas, in south-western Uganda, were invited to participate in the study. Face-to-face recruitment generated high response rate of 93% and thus few have been missed. In this study some assistants in medical laboratories, pharmacies and nursing were enrolled as well because they were part of the diabetes management providers. The study population had characteristics similar to what is found in Uganda in an area outside the capital city [15]. Therefore it is argued that the sample is representative and the results can be generalized [23]. Further, the intention of the study was to generate primary information from healthcare professionals who provide diabetes care, which is a strength.

## Conclusion

Healthcare providers reported being knowledgeable about diabetes care and its symptoms. Some of the modifiable risk factors affecting management of diabetes and its prevention were outlined to be lack of awareness of signs and symptoms of diabetes among people with diabetes, compromised healthcare-seeking practices such as self-medication, procurement of diabetes drugs and seeking care from traditional, spiritual healers when their expectations were not met by the professional health sector. Co-morbidities among patients with diabetes were reported to be dominated by symptoms or complications of diabetes related to poor glycaemic control. Because of strained economic resources and a poor healthcare system, most of the patients in Africa are diagnosed only after diabetes has advanced with symptoms or complications [34]. Care actors from the popular, folk and professional health sectors need to co-operate, share knowledge and develop a systematic referral system since healthcare providers seem to have positive attitude towards CAM. Well-organized diabetes care is urgently needed, dedicated to diabetes education, raising awareness about diabetes and its severity and self-care management to achieve and maintain glycaemic control in order to prevent or delay the development of costly complications. The costs associated with diabetes include increased use of health services, productivity loss, and disability, which can be a considerable burden to the individual, families and the nation [10].

### What is known about this topic

- Healthcare-seeking behaviour among persons with diabetes has been investigated to a limited extent in developing countries, and not from health professionals’ perspective;
- Healthcare-seeking behaviour with a switch between different healthcare providers under the influence of the popular and folk sector has been reported from the perspectives of the persons with diabetes and traditional healers. The switch may negatively affect diabetes management and health.

### What this study adds

- Most of the healthcare providers perceived more uneducated people to be at risk of developing complications related to diabetes and that most of the patients with diabetes were not knowledgeable about signs and symptoms of diabetes before being diagnosed;
- The main reasons inducing persons with diabetes to seek care outside the health care sector were reported to be seeking a cure for the condition, influence from the popular sector, the accessibility of the place and signs of complications of diabetes related to poor glycaemic control;
- Healthcare providers had relatively positive attitudes towards using complementary and alternative medicine.

## Competing interests

The authors declare no competing interests.

## References

[1] Hjelm K, Atwine F (2011). Health-care seeking behaviour among persons with diabetes in Uganda: an interview study. BMC International Health and Human Rights..

[2] Atwine F, Hultsjo S, Albin B, Hjelm K (2015). Health-care seeking behaviour and the use of traditional medicine among persons with type 2 diabetes in south-western Uganda: a study of focus group interviews. Pan African Medical Journal..

[3] Atwine F, Hjelm K (2016). Healthcare-seeking behaviour and management of type 2 diabetes: from Ugandan traditional healers’ perspective. International Journal of African Nursing Sciences..

[4] Kalyango NJ, Owino E, Nambuya PA (2008). Non-adherence to diabetes treatment at Mulago hospital in Uganda: prevalence and association factors. African Health Sciences..

[5] American Diabetes Association (ADA) (2015). Diabetes care: Standards of medical care in diabetes-201. The Journal of Clinical and Applied Research and Education..

[6] Kleinman A (1980). Patients and healers in the context of culture: Comparative studies of health systems and medical care.

[7] World Health Organization (2013). Traditional Medicine Strategy 2014-2023.

[8] Lie De, Boker J (2004). Development and validation of the CAM Health Belief Questionnaire (CHBQ) and CAM use and attitudes amongst medical students. BMC Medical Education..

[9] Kumar D, Bajaj S, Mehrotra R (2006). Knowledge, attitude and practice of complementary and alternative medicines for diabetes. Public Health..

[10] IDF (2015). Epidemiology & research, IDF Diabetes Atlas..

[11] Guariguata L, Whiting RD, Hambleton I, Beagley J, Linnenkamp U, Shaw EJ (2014). IDF Diabetes Atlas: global estimates of diabetes prevalence for 2013 and projection for 2035. Diabetes Research and Clinical Practice..

[12] Mbanya JCM, Motal A, Sobngwi E, Assah KF, Enoru TS (2010). Diabetes in Sub-Saharan Africa. Lancet.

[13] Peer N, Kengne A, Motala AA, Mbanya JC (2014). IDF Diabetes atlas - Diabetes in the African region: an update. Diabetes Research and Clinical Practice..

[14] Ministry of Health (2010). Uganda Health Sector Strategic & Investment Plan 2010/11-2014/15. Promoting people’s health to enhance socio-economic development..

[15] Mukasa N (2012). Uganda Healthcare system profile: Background, Organization, Polices and Challenges. Journal of Sustainable Regional Health Systems..

[16] Chang H, Chang H (2015). A Review of nurses’ knowledge, attitudes and ability to communicate the risk and benefits of complementary and alternative medicine. Journal of Clinical Nursing..

[17] Shorofi A S, Arbon P (2010). Nurses’ knowledge, attitudes and professional use of complementary and alternative medicine (CAM): A survey at five metropolitan hospitals in Adelaide. Complementary Therapies in Clinical Practice..

[18] Adib-Hajbaghery M, Hoseinian M (2014). Knowledge, attitude and practice toward complementary and traditional medicine among Kashan health care staff, 2012. Complement Ther Med..

[19] Zhang Y, Peck K, Spalding M, Xu T, Ragain M (2010). A study to examine the attitudes, knowledge, and utilization of CAM by primary care professional in West Texas. Complement Ther Med..

[20] Samuels N, Zisk-Rony Y R, Many A, Ben-Shitrit G, Erez O, Mankuta D, Rabinowitz R, Lavie O, Shuval TJ, Oberbaum M (2013). Use of and attitudes towards complementary and alternative medicine among obstetricians in Israel. International Journal of Gynecology and Obstetrics..

[21] Orkaby B, Greenberger C (2015). Israeli Nurses’ attitudes to the holistic approach to health and their use of complementary and alternative therapies. Journal of Holistic Nursing..

[22] Haas L, Maryniuk M, Beck J, Cox CE, Duker P, Edwards Fisher BE (2013). National standards for diabetes self-management education and support. Diabetes Care..

[23] Polit DE, Beck TC (2008). Nursing Research: Generating and Assessing Evidence for Nursing Practice ed 8.

[24] Nguma LK (2010). Health seeking and health related behaviour for type 2 diabetes mellitus among adults in an urban community in Tanzania.

[25] Rosenstock IM, Strecher JV, Becker HM (1988). Social learning theory and the Health Belief Model. Health Educ Q.

[26] Kroeger A (1983). Anthropological and socio-medical health care research in developing countries. Social Science and Medicine..

[27] World Medical Association (WMA) (2013). Declaration of Helsinki: Ethical Principles for Medical Research involving Human Subject. Journal of the American Medical Association..

[28] Altman Douglas G (1991). Practical statistics for medical research.

[29] Inzucchi SE, Bergenstal RM, Buse JB, Diamant M, Ferrannini E, Nauck M, Peters A L, Tsapas A, Wender R, Matthews DR (2012). Management of hyperglycemia in type 2 diabetes: a patient-centred approach. Diabetes Care..

[30] Peck R, Mghamba J, Vanobberghen F, Kavish B, Rugarabamu V, Smeeth L, Hayes R, Grosskurth H, Kapiga S (2014). Preparedness of Tanzanian health facilities for outpatient primary care of hypertension and diabetes: a cross-sectional survey. Lancet Glob Health..

[31] Schwartz I J, Guwatudde D, Nugent R, Kiiza M C (2014). Looking at non-communicable diseases in Uganda through a local lens: an analysis using locally derived data. Global Health..

[32] Kanungo S, Bhowmik K, Mahapatra T, Mahapatra S, Bhadra UK, Sarkar K (2015). Perceived morbidity, health-care-seeking behavior and their determinants in a poor-resource setting: observation from India. PLoS One..

[33] Katende D, Mutungi G, Baisley K, Biraro S, Ikoona E, Peck R, Smeeth L, Hayes R, Munderi P, Grosskurth H (2015). Readiness of Ugandan health services for the management of outpatients with chronic diseases. Trop Med Int Health..

[34] Tuei CV, Maiyoh KG, Ha C (2010). Type 2 diabetes and obesity in sub-Saharan Africa. Diabetes/Metabolism Research and Reviews..

